# Light Stress-Induced Increase of Sphingosine 1-Phosphate in Photoreceptors and Its Relevance to Retinal Degeneration

**DOI:** 10.3390/ijms20153670

**Published:** 2019-07-26

**Authors:** Ryo Terao, Megumi Honjo, Takashi Ueta, Hideru Obinata, Takashi Izumi, Makoto Kurano, Yutaka Yatomi, Hideto Koso, Sumiko Watanabe, Makoto Aihara

**Affiliations:** 1 Department of Ophthalmology, Graduate School of Medicine, Tokyo University, Tokyo 113-8654, Japan; 2 Gunma University Initiative for Advanced Research (GIAR), 3-39-22 Showa-machi, Maebashi, Gunma 371-8511, Japan; 3 Department of Biochemistry, Gunma University Graduate School of Medicine, 3-39-22 Showa-machi, Maebashi, Gunma 371-8511, Japan; 4 Department of Clinical Laboratory Medicine, The University of Tokyo, 7-3-1, Hongo, Bunkyo-ku, Tokyo 113-8654, Japan; 5 Division of Molecular and Developmental Biology, Institute of Medical Science, University of Tokyo, 4-6-1 Shirokanedai, Minato-ku, Tokyo 108-8639, Japan

**Keywords:** sphingosine 1-phosphate, sphingolipids and inflammation, light-induced retinal degeneration, choroidal neovascularization, age-related macular degeneration, visual cycle, sphingosine kinase

## Abstract

Sphingosine 1-phosphate (S1P) is a potent lipid mediator that modulates inflammation and angiogenesis. In this study, we investigated the possible involvement of S1P in the pathology of light-induced retinal degeneration in vivo and in vitro. The intracellular S1P and sphingosine kinase (SphK) activity in a photoreceptor cell line (661W cells) was significantly increased by exposure to light. The enhancement of SphK1 expression was dependent on illumination, and all-trans-retinal significantly promoted SphK1 expression. S1P treatment reduced protein kinase B (Akt) phosphorylation and increased the protein expression of cleaved caspase-3, and induced photoreceptor cell apoptosis. In vivo, light exposure enhanced the expression of SphK1 in the outer segments of photoreceptors. Intravitreal injection of a SphK inhibitor significantly suppressed the thinning of the outer nuclear layer and ameliorated the attenuation of the amplitudes of a-waves and b-waves of electroretinograms during light-induced retinal degeneration. These findings imply that light exposure induces the synthesis of S1P in photoreceptors by upregulating SphK1, which is facilitated by all-trans-retinal, causing retinal degeneration. Inhibition of this enhancement may be a therapeutic target of outer retinal degeneration, including age-related macular degeneration.

## 1. Introduction

Age-related macular degeneration (AMD) is one of the most common vision-threatening diseases in the world. Various factors, including age, environmental factors, and genetic factors, have been implicated as causes of AMD [1,2,3], but the pathogenesis is relatively unknown. One of the most important factors associated with the pathology of AMD is retinal damage induced by light. Numerous studies have reported that excessive light exposure leads to lipid peroxidation and retinal damage, which causes the pathogenesis of AMD [4,5]. A light-induced retinal degeneration model has been used as an animal model for AMD [6,7,8]. However, the detailed mechanism of light-induced retinal damage remains unknown.

Sphingosine 1-phosphate (S1P) is a bioactive lipid mediator that is synthesized via the phosphorylation of sphingosine by sphingosine kinase (SphK, SphK1, and SphK2). S1P helps regulate various cellular processes, including angiogenesis, cell proliferation, migration, inflammation, and cell death [9,10,11,12]. S1P is also the ligand for five G protein-coupled receptors (S1P1–5), which mediate cellular processes by activating GTPases, such as Rho and Rac. We previously reported that S1P is associated with angiogenic and inflammatory responses in retinal pigment epithelial (RPE) cells [13,14], and proposed that inhibition of the interaction between S1P and S1P receptor, or treatment with a carrier of S1P, apolipoprotein M, may be a novel treatment strategy for choroidal neovascularization. However, we did not clarify the presence and activation dynamics of S1P in the retina. A report recently characterized the expression of SphKs and S1P receptors in rat and murine eyes, and found that the expression of SphK1, S1P2, and S1P3 in the retina is upregulated by light [15]. The findings strongly implied that S1P is associated with cell death and retinal degeneration in the mechanism of light-induced retinal degeneration. In the present study, we showed that light exposure affected S1P and SphK1 upregulation in retinal photoreceptor cells, both in vivo and in vitro. Additionally, we demonstrated that inhibition of S1P had a protective effect on photoreceptors in a retinal degeneration murine model induced by light exposure.

## 2. Results

### 2.1. Light-Emitting Diode Exposure Enhanced S1P in Photoreceptor Cells

**Figure 1:**
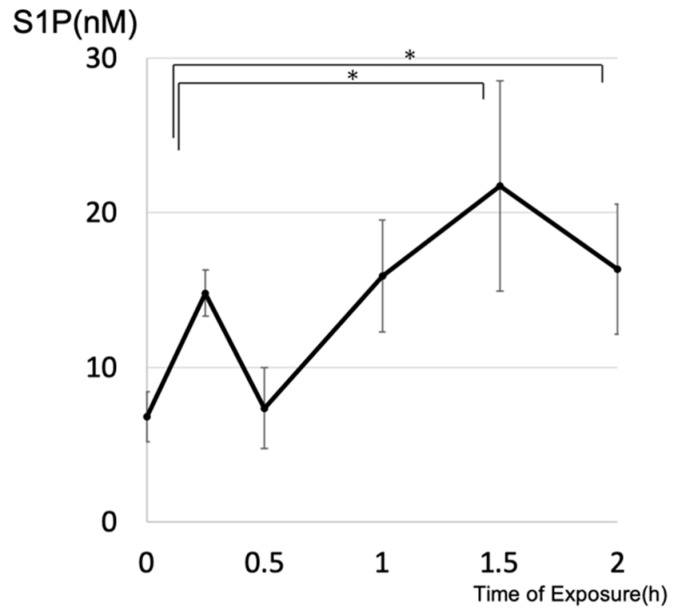
Light exposure increased intracellular sphingosine 1-phosphate in photoreceptors. 661W cells were exposed to a light-emitting diode (approximately 8000 lux) (*n* = 4, each group). Data are presented as mean ± standard error. Statistical analyses were performed using Dunnett’s test. * Significant *p*-values are labeled with *p* < 0.05.

**Figure 2:**
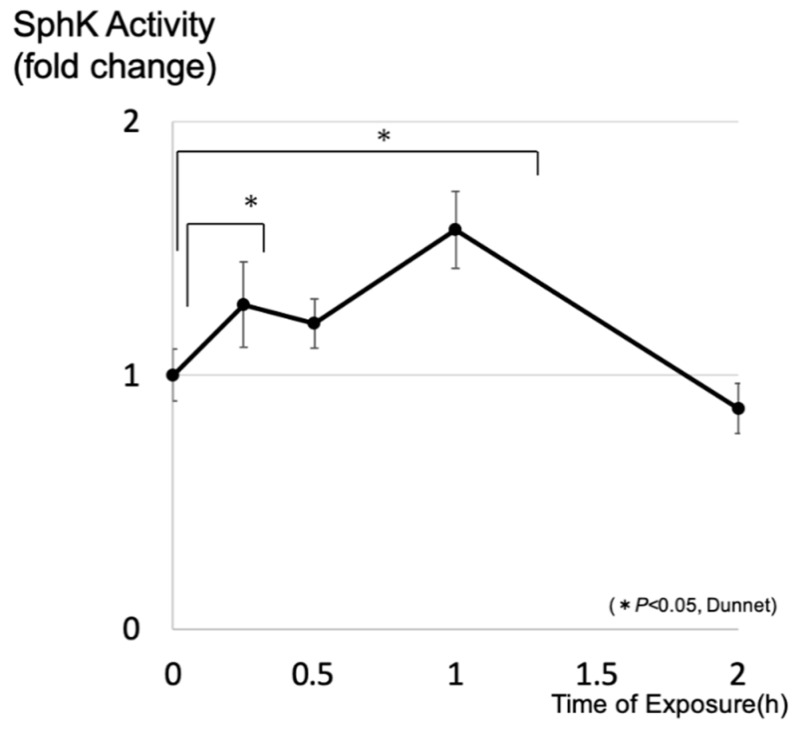
Light exposure enhanced the sphingosine kinase (SphK) activity of photoreceptor cells. 661W cells were exposed to a light-emitting diode (approximately 8000 lux). SphK activity was evaluated using a SphK activity assay (*n* = 4, each group). Data are presented as a mean ± standard error. Statistical analysis was performed using Dunnett’s test. * Significant *p*-values are labeled with (*p* < 0.05).

After exposure to a light-emitting diode (LED), the concentrations of intracellular S1P in cultured 661W cells were determined by liquid chromatography–mass spectrometry (LC-MS). A time-course study showed that intracellular levels of S1P increased significantly by 1.5 h after treatment, which indicates that light stimulation of photoreceptor cells induced enhancement of intracellular S1P (Figure 1). We also investigated whether light exposure affected the activity of SphK, which is a key enzyme that synthesizes S1P from sphingosine. Light exposure significantly increased the SphK activity of photoreceptor cells within 1 h after exposure, preceding the increase of S1P (Figure 2).

**Figure 3:**
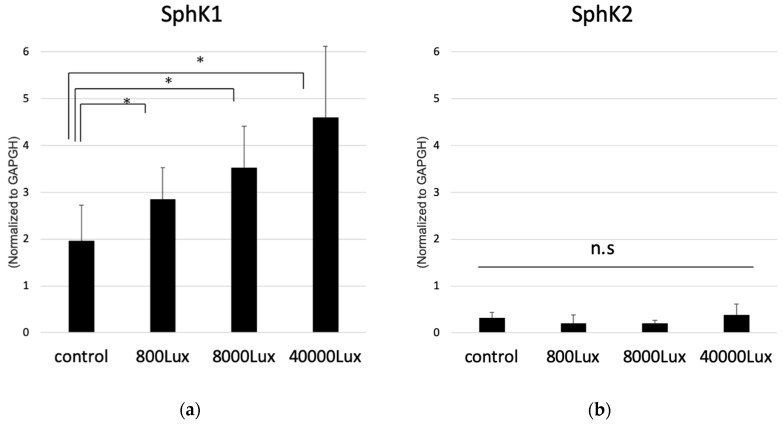
Expression of sphingosine kinase (SphK) 1 (**a**) and 2 (**b**) in photoreceptor cells detected by the quantitative polymerase chain reaction normalized to the housekeeping gene of glyceraldehyde-3-phosphate dehydrogenase (GAPDH). 661W cells were exposed to a light-emitting diode with various intensities (800 lux, 8000 lux, and 40,000 lux) (*n* = 6, each group). Each column represents the mean ± standard error. Statistical analysis was performed using Dunnett’s test. * Significant *p*-values are labeled with *p* < 0.05. n.s: not significant.

We then determined whether the illumination intensity of the LED-affected SphK expression. Quantitative real-time polymerase chain reaction (qPCR) analysis showed that expression of SphK1 mRNA was significantly increased in an illuminance-dependent manner (Figure 3). However, SphK2 expression was much lower than that of SphK1, and did not show any significant change with light exposure (Figure 3), which indicated that SphK1 was the dominant enzyme that synthesized S1P in photoreceptor cells, and that light stimulation enhanced the expression of SphK1 as a function of light intensity. These results collectively imply that light exposure enhanced the expression of SphK, especially SphK1, which resulted in an increase of intracellular S1P in photoreceptors.

### 2.2. All-Trans Retinal Induces the Expression of Sphingosine Kinase (SphK) in Photoreceptor Cells

**Figure 4:**
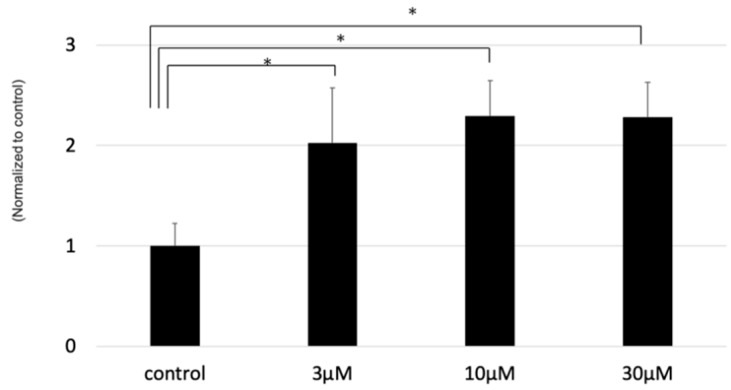
The effect of all-trans-retinal (atRAL) on the expression of sphingosine kinase (SphK) 1 in photoreceptor cells (661W cells) as detected by the quantitative polymerase chain reaction. Samples were collected one hour after treatment with atRAL (*n* = 6, each group). Expression of SphK1 was normalized to the housekeeping gene of glyceraldehyde-3-phosphate dehydrogenase. Each bar showed the mean ± standard error of the ratio to the control. Statistical analysis was performed using Dunnett’s test. * Significant p-values are labeled with *p* < 0.05.

As the next step, we further researched the mechanism for enhancing SphK1 in photoreceptors by light exposure. All-trans-retinal (atRAL) is an intermediate of the visual cycle, which is known to be released in the rod outer segment (OS) by photoactivated rhodopsin after light excitation, and has a toxic effect on photoreceptors [16]. Therefore, atRAL is reportedly thought of as one that causes light-induced retinal degeneration [17]. We hypothesized that atRAL may affect the dynamics of sphingolipids in the retina, and examined the bioactive effect of atRAL on sphingolipid enzymes in photoreceptor cells. As a result, atRAL significantly enhanced the expression of SphK1 in photoreceptors in a dose-dependent manner (Figure 4). This result affirmed that the light-induced upregulation of atRAL may enhance the expression of SphK1, causing the increased S1P in the retina.

### 2.3. Light Exposure Increases the Expression of SphK1 in Murine Retina

**Figure 5:**
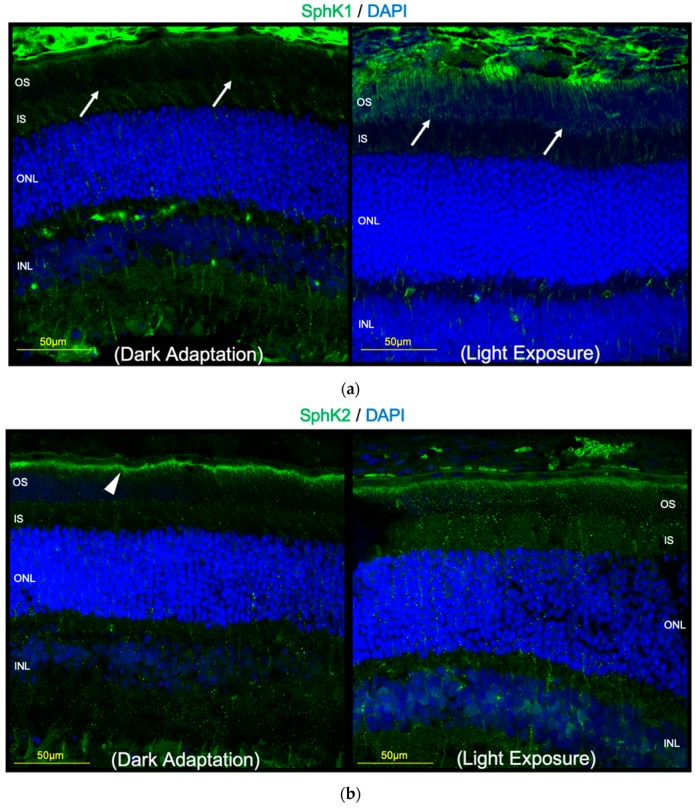
A mouse was exposed to light (approximately 8000 lux) for 1 h and then enucleated. Images represent immunohistochemistry of sphingosine kinase (SphK) (**a**) 1 and (**b**) 2, as well as 4′,6-diamidino-2′-phenylindole dihydrochloride. Light exposure enhanced SphK1 expression in the outer segments of murine photoreceptors (arrow). SphK2 expression was concentrated in the retinal pigment epithelium microvilli (arrowhead), and was not affected by light exposure. Bar, 50 µm.

We also investigated whether light exposure affects the expression of SphK1 or S1P in retinal tissues in vivo. As previously reported, the expression of SphK was reportedly seen throughout the retina [15]. Our study also found that SphK1 and SphK2 was expressed all around the retinal tissues (Figure 5). In addition, we investigated the influence of light exposure on the expression of SphK in the retina. As a result, we found that the light exposure increased the expression of SphK1 in OS of the photoreceptor (Figure 5A). The expression of SphK2 seemed to be particularly localized in RPE microvilli, but did not change significantly after the light exposure. These in vivo results support our in vitro results that SphK1-S1P upregulation could be crucial in the light-induced degeneration, possibly as a downstream of atRAL in the light reception activity in photoreceptors.

### 2.4. S1P Induced the Apoptosis of Photoreceptor Cells

**Figure 6:**
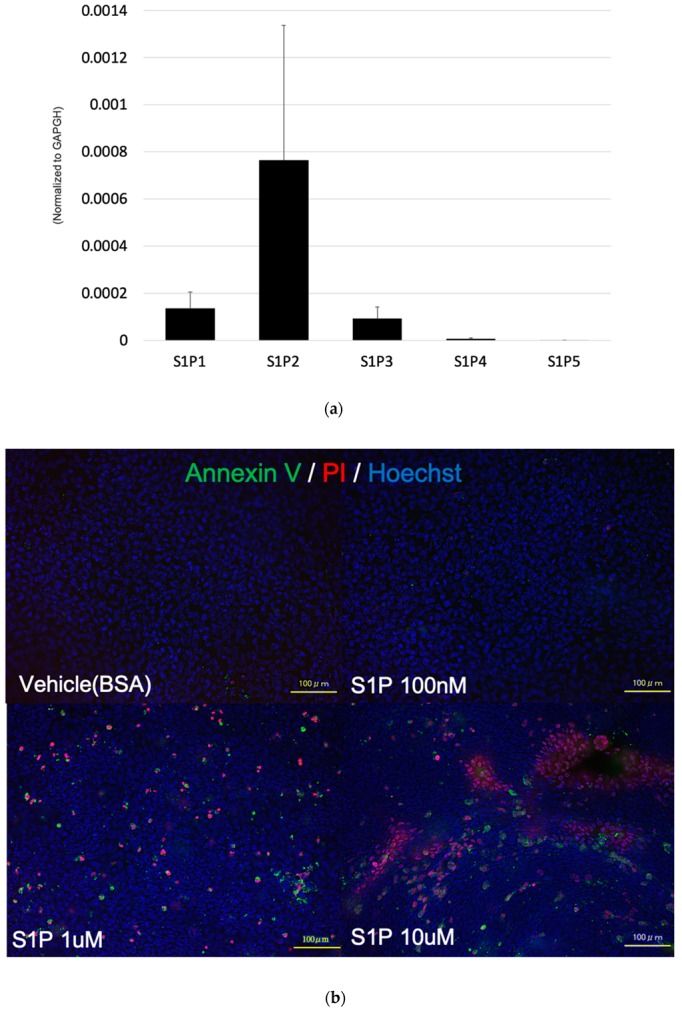

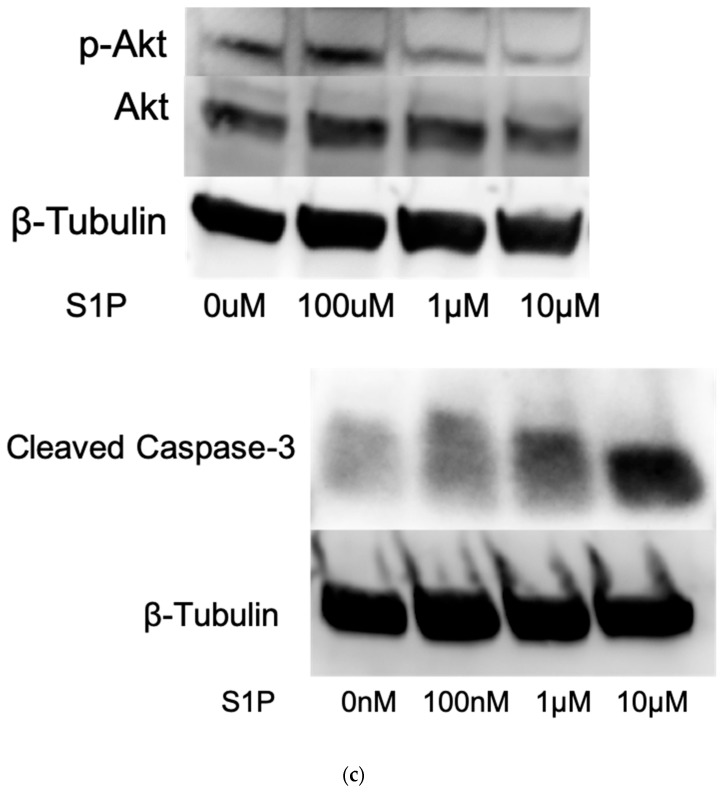
Sphingosine 1-phosphate (S1P) induced apoptosis of photoreceptor cells. (**a**) The expression of S1P receptors in 661W cells detected by the quantitative polymerase chain reaction was normalized to the housekeeping gene of glyceraldehyde-3-phosphate dehydrogenase (GAPDH) (*n* = 6). Each column represents the mean ± standard error. (**b**) 661W cells were treated with S1P for 7 h, which was followed by staining with the annexin V/propidium iodide, and Hoechst 33342. Bar: 100 µm. (**c**) Western blot analysis showing phospho-Akt, Akt, cleaved capase-3, and β-tubulin.

To determine the effect of S1P on photoreceptor cell viability, we evaluated the expression of S1P receptors by using qPCR. As a result, the expression of S1P2 was the highest of all S1P receptors, whereas that of S1P4 and S1P5 were rarely seen in 661W cells (Figure 6A). As a next step, we performed annexin V/propidium iodide staining, and found that the number of apoptotic cells increased after treatment with 1 μM or 10 μM S1P (Figure 6B). Treatment with vehicle (lipid-free bovine serum albumin) or 100 nM S1P did not affect photoreceptor apoptosis. Western blotting to detect the expression of apoptotic protein showed that S1P decreased Akt phosphorylation and increased the expression of cleaved caspase-3 in photoreceptor cells (Figure 6C). Taken together, these results suggested that increased S1P triggered apoptosis in photoreceptor cells by inactivating Akt and increasing cleaved caspase-3, which caused retinal degeneration.

### 2.5. A SphK Inhibitor Conferred a Protective Effect against LED Light-Induced Retinal Degeneration

**Figure 7:**
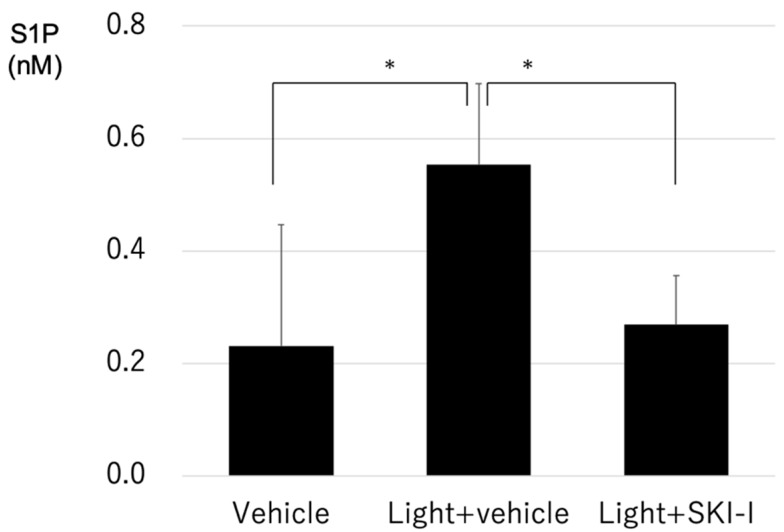
Light exposure increased intraretinal sphingosine 1-phosphate in mice. Mice were exposed to a light-emitting diode light (approximately 8000 lux for 1.5 h and 24 h) after intravitreal injection of a SphK inhibitor (SKI-I) or vehicle (dimethyl sulfoxide) (Vehicle *n* = 19, Light + vehicle *n* = 5, Light + SKI-I *n* = 5). Each column represents the mean ± standard error. Statistical analysis was performed using the unpaired *t*-test. * Significant *p*-values are labeled with *p* < 0.05.

Based on the previous results, we hypothesized that light exposure enhanced S1P in photoreceptors, which induced apoptosis of photoreceptors. To confirm our hypothesis, we determined whether the inhibition of SphK and resulting S1P suppression attenuated light-induced retinal degeneration. First, we measured the concentration of S1P in murine retinas, and found that light exposure significantly increased intraretinal S1P, and that the SphK1 and SphK2 dual inhibitor (SKI-I) significantly decreased the enhancement of S1P in murine retinas (Figure 7).

**Figure 8:**
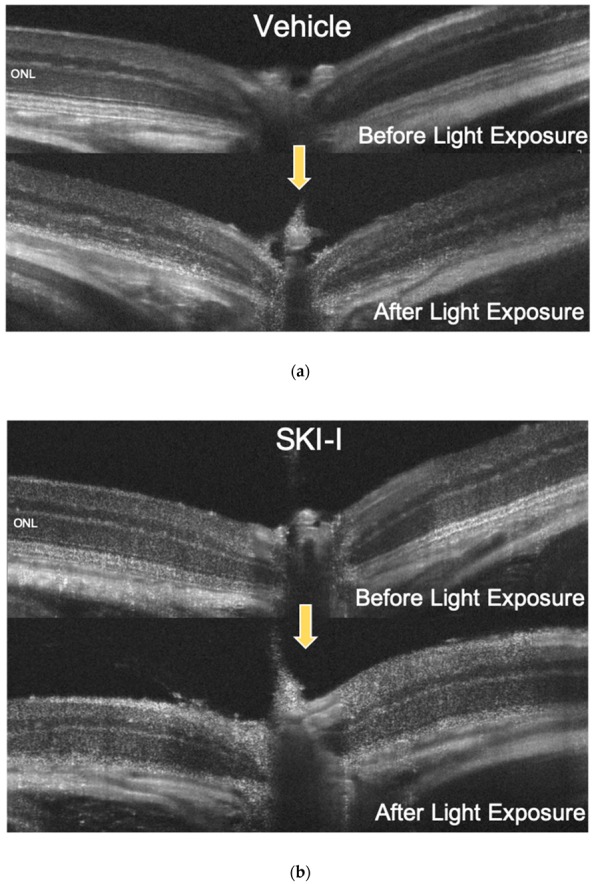

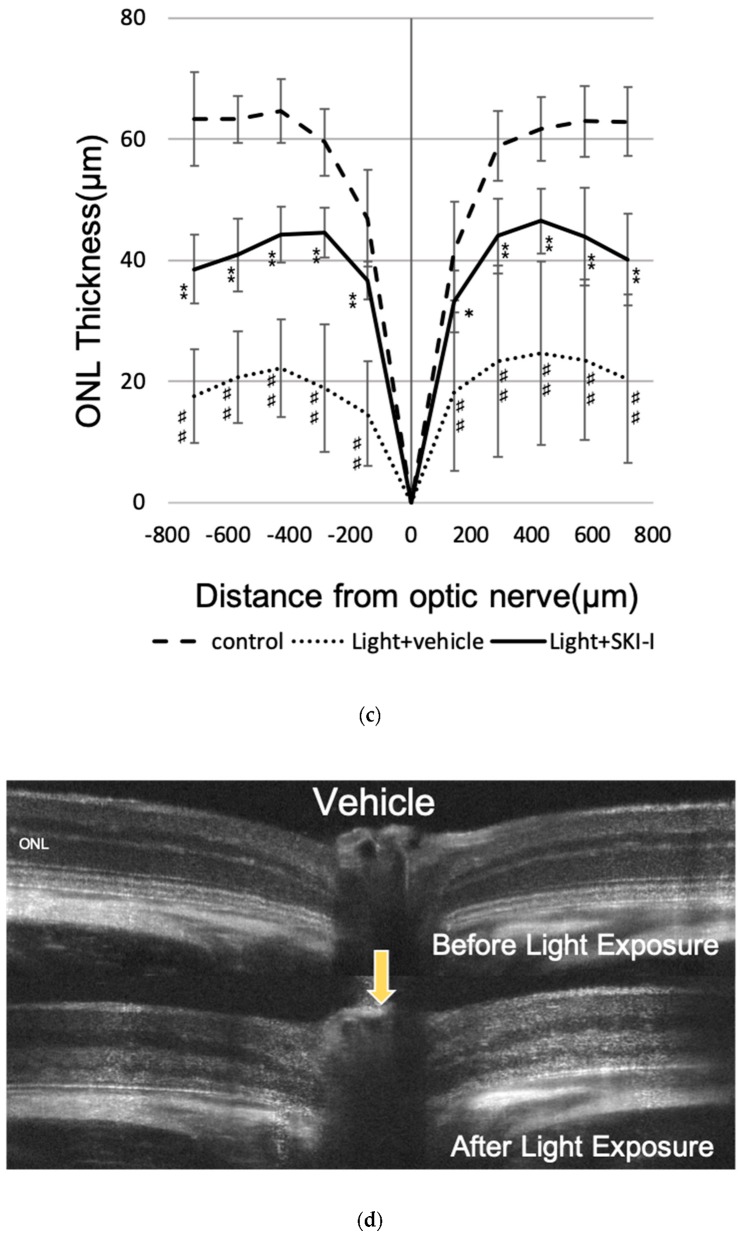

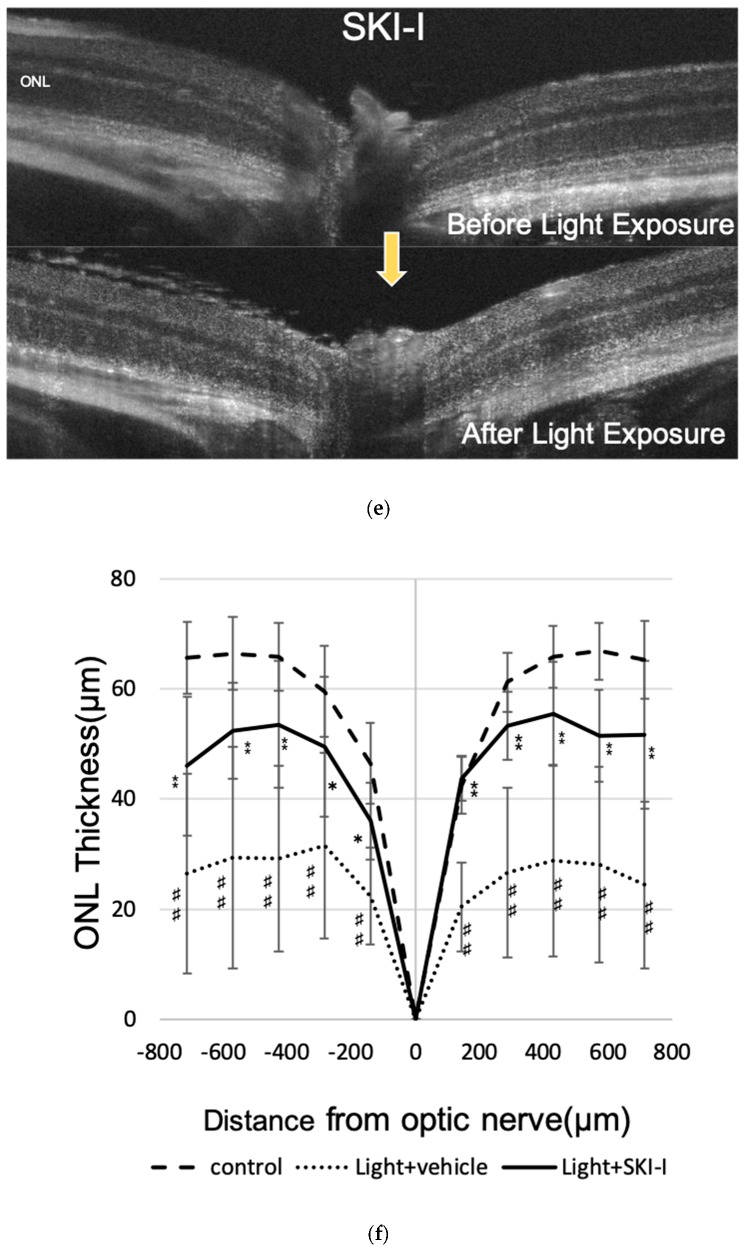

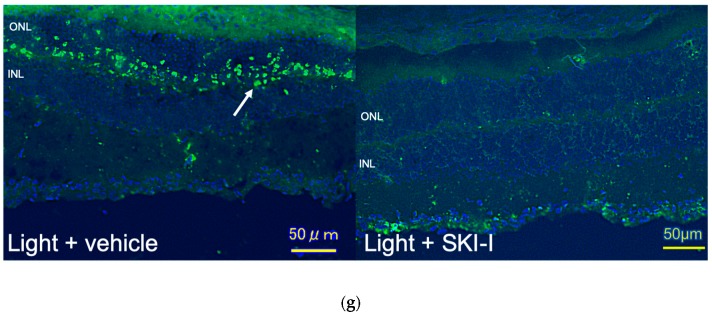
Inhibition of sphingosine kinase (SphK) suppressed light-induced murine retinal degeneration. Mice were administered with vehicle (dimethyl sulfoxide, DMSO) in one eye (**a**), and the other eye was treated with SphK inhibitor (SKI-I) intravitreally (**b**). Optical coherence tomography (OCT) images of murine retina were obtained before and five days after light exposure to a light-emitting diode (LED, approximately 8000 lux for 4 h). (**c**) Measurement of the outer nuclear layer thickness (*n* = 6, each group). (**d**,**e**) OCT images of murine retinas before and five days after light exposure using an LED (approximately 30,000 lux for 4 h). One eye in each mouse was treated with vehicle (DMSO) (**d**), and the other eye was treated with SKI-I intravitreally (**e**). (**f**) Measurement of the outer nuclear layer thickness (*n* = 5, each group). (**g**) Retinal sections were stained using the TUNEL assay. Apoptotic photoreceptors were observed in vehicle-treated mice after the light exposure with the LED (approximately 8000 lux for 4 h, arrow). Each column represents the mean ± standard error. Statistical analysis was performed using analysis of variance and the Tukey–Kramer post hoc test * *p* < 0.05, ⁑ *p* < 0.01 (vs. light + vehicle), ^♯^ *p* < 0.05, ^♯♯^ *p* < 0.01 (vs. vehicle). Bar: 50 µm.

**Figure 9:**
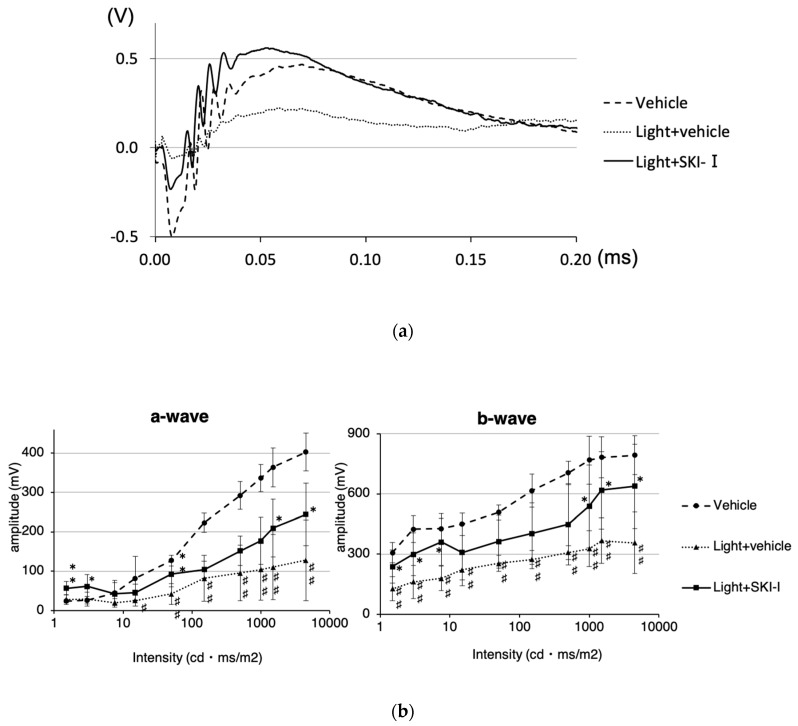
The effect of a sphingosine kinase inhibitor on the retinal function of light-induced murine retinal degeneration. An electroretinogram (ERG) was performed four days after light exposure (Vehicle *n* = 6, Light + vehicle *n* = 9, Light + SKI-I *n* = 8). (**a**) Representative ERG responses. (**b**) Amplitudes of a-waves and b-waves. Each column represents the mean ± standard error. Statistical analysis was performed using the analysis of variance and the Tukey–Kramer post hoc test. * *p* < 0.05, ⁑ *p* < 0.01 (vs. light + vehicle), ^♯^ *p* < 0.05, ^♯♯^ *p* < 0.01 (vs. vehicle).

We also measured the thickness of the outer nuclear layer (ONL) to assess the retinal damage using optical coherence tomography (OCT). Light damage (approximately 8000 lux or 30,000 lux for 4 h) significantly decreased the ONL thickness. However, intravitreal injection of the SphK inhibitor significantly suppressed ONL thinning (Figure 8A–D). In addition, terminal deoxynucleotidyl transferase–mediated biotinylated UTP nick end labeling (TUNEL) staining showed that SphK1 and SphK2 inhibition restrained apoptosis of photoreceptor cells compared to the control (Figure 8E). These results suggested that the increased activity of SphK, promoted by light exposure, inhibited light-induced photoreceptor apoptosis, but the inhibition of SphK showed a suppressive effect on photoreceptor cell death. We also evaluated the effect of the SphK inhibitor on retinal function attenuated by light damage. Excess light exposure (8000 lux for 4 h) significantly decreased the amplitude of both a-waves and b-waves in electroretinograms (ERGs). Intravitreal injection of the SphK inhibitor suppressed the reduction of a-wave and b-wave amplitudes (Figure 9A). At 4500 cdms/m^2^, administration of the SphK inhibitor significantly improved the a-wave and b-wave amplitudes by 42.1% and 64.6%, respectively (Figure 9B). Together, these results showed that treatment with the SphK inhibitor restrained both morphological and functional retinal failure induced by light damage, which indicated the crucial role of S1P in light-induced retinal degeneration.

## 3. Discussion

The purpose of the present study was to clarify whether S1P is involved in the pathology of light-induced retinal degeneration. Numerous reports have documented the crucial role of excessive light in the pathology of AMD [6,7,8]. We characterized the effect of light exposure on the production of S1P in photoreceptors, and found that intracellular S1P in photoreceptor cells increased when exposed to LED light. This biological change in cultured photoreceptor cells was attributed to the upregulation of SphK1 induced by light, which was significantly increased in an illuminance-dependent manner. This implies a connection between S1P and the pathology of retinal degeneration.

We also characterized the in vivo expression of SphK in murine retinas. Porter et al. [15] reported that both SphK1 and SphK2 expression were localized throughout the rat retina. They found that SphK1 expression was especially concentrated in ganglion cell bodies, the inner plexiform layer, and the ONL. SphK2 expression was especially concentrated in the ganglion cell layer, inner nuclear layer, ONL, and the RPE layer. In the present study, we also observed the localization of SphK1 and SphK2 throughout the murine retina, and found that light stimulation enhanced the expression of SphK1 in the OSs of photoreceptors. The OS includes unique organelles specialized for light perception, and possesses stacked membrane discs containing rhodopsin and other proteins, which are important for phototransduction [18]. Our finding of the enhancement of SphK1 expression in the OS by light indicated that SphK1 was associated with light absorption in photoreceptors.

Based on our results that light exposure enhanced the expression of SphK in photoreceptors, we hypothesized that the regulation of SphK was associated with light reception of the OSs of photoreceptors. Therefore, we focused on the visual cycle as a possible mechanism of light illuminance-dependent SphK enhancement. The visual cycle involves the conversion of a single photon of light energy into an electrical signal in the retina [19]. It bleaches and recycles retinoids for the synthesis of visual pigments among photoreceptor OSs and RPE cells. The vertebrate vision begins with light absorption by rhodopsin. When exposed to light, 11-cis-retinal, the chromophore of rhodopsin, is photo-isomerized into atRAL in photoreceptors, which is liberated from rhodopsin and converted into all-trans-retinol (vitamin A). Then, atRAL is transported to RPE cells, where 11-cis-retinal is regenerated. Previous studies have found that atRAL has cytotoxic effects on photoreceptors and RPE cells, which results in the accumulation of N-retinylidene-N-retinylethanolamine (A2E) and the pathogenesis of retinal diseases, including Stargardt disease, AMD, and light-induced retinal degeneration [20]. A mutation of *ABCA4*, encoding the transporter of atRAL to the outside of discs, has been shown to cause Stargardt disease and recessive retinal pigmentosa [21,22,23]. In the present study, we hypothesized that SphK1 and S1P were involved in the pathophysiology of retinal downstream of atRAL, and found that atRAL increased the expression of SphK1 in photoreceptor cells in a dose-dependent manner. This result identified one of the mechanisms of light illuminance-dependent increases of S1P, which involved the increase of SphK1 in photoreceptors after light exposure as a result of enhanced endogenous atRAL. We further investigated the biochemical effect of S1P on photoreceptors. Because atRAL induces photoreceptor cell death via caspase activation and has a strong association with the pathology of light-induced retinal degeneration [24], we hypothesized that S1P was also associated with photoreceptor cell death and retinal degeneration as a downstream signal of atRAL. Numerous reports have established the cellular proliferative and anti-apoptotic effects of S1P on various tissues, especially in malignant neoplasms. Guzel et al. [25] reported that exogenous S1P inhibited apoptosis of human ovarian follicles in vitro. In the present report, S1P was used at 200 and 400 μM concentrations. Zeng et al. [26] also demonstrated that 2 μM S1P suppressed hepatocellular carcinoma cell apoptosis via syndecan-1. Regarding photoreceptors, there have been very few reports describing the association of S1P with cell viability. A previous study reported that S1P increased the expression of opsin, and was necessary for the differentiation of photoreceptor cells [27]. In the present report, cultured rat photoreceptor progenitor cells were treated with albumin-bound S1P, to show that 1 μM S1P significantly reduced the number of apoptotic progenitor cells. Wilkerson et al. [28] reported that SphK1 knockout mice presented with disrupted OSs and inner segments of photoreceptors. Fabiani et al. [29] found that S1P lyase inhibitor suppressed H_2_O_2_-induced photoreceptor cell (661W cells) death. These reports confirmed the role of S1P as a key molecule in the maintenance of homeostasis and viability of photoreceptors during tissue development and other physiological processes. Stiles et al. [30] reported that systemic administration of FTY720 (fingolimod, a S1P receptor modulator) suppressed photoreceptor degeneration in P23H-1 rats, which is a rat retinal degeneration model. In this report, intraretinal S1P in P23H-1 rats was much higher than that of control rats, and administration of FTY720 reduced intraretinal S1P to levels closer to that of normal rats. Chen et al. [31] also reported that inhibition of ceramide synthesis suppressed light-induced retinal degeneration in albino rats via FTY720 administration. In the same report, light exposure significantly increased the concentration of S1P in rat retinas. In the present study, we also found that the concentration of S1P in murine retinas was significantly increased by light exposure, which was confirmed by LC-MS (Figure 7). These reports supported our present results that S1P was also upregulated in a retinal degeneration model, which indicated that S1P is associated with photoreceptor degeneration.

Moreover, there has been no study investigating the direct effect of S1P on the viability of photoreceptor cells. As shown in Figure 6, we found that S1P was responsible for an apoptotic signal in photoreceptor cells. Consistent with this observation, some reports have suggested an association between S1P synthesis and apoptosis. Liu et al. [32] reported that overexpression of SphK2 induced apoptosis in a variety of cells via cytochrome c release and caspase-3 activation. Igarashi et al. [33] demonstrated that SphK2, which exists in the nuclei, inhibited DNA synthesis. According to other reports, these apoptotic and antiproliferative effects of S1P are induced by the inactivation of the Akt pathway, which is involved in cell proliferation and survival. Kim et al. reported that the treatment with S1P inactivated Akt/protein kinase B, which results the suppression of keratinocyte proliferation [34]. Schuppel et al. described that S1P suppressed Insulin-mediated keratinocyte proliferation by reducing phosphorylation of Akt via S1P2 [35]. Considering Akt downregulates the activity of caspase-3 [36], it is crucial for apoptosis to suppress Akt phosphorylation. Our results regarding the distribution of S1P receptors found that the expression of S1P2, which is presumably associated with apoptosis, was the highest (Figure 6A). Although our results suggested that SphK1, and not SphK2, was dominantly upregulated by light exposure (Figure 3), we also confirmed that exogenous S1P enhanced photoreceptor apoptosis by inactivating Akt, which leads to activation of cleaved caspase-3 (Figure 6). Collectively, the results showed that S1P, which was augmented by light exposure and possibly synthesized via SphK1 in photoreceptors, induced photoreceptor death. This resulted in retinal dysfunction. These results encouraged us to evaluate the role of SphK, and confirmed the effect of its inhibition in the cell viability of photoreceptors in subsequent experiments.

Next, we evaluated the role of SphK1 and the resulting suppression of S1P synthesis in light-induced retinal degeneration in mice using a SphK inhibitor. We used 8000 lux LED light stimulation to determine the effect of the suppression of S1P synthesis under pathologically strong light exposure. Figure 7 shows that the light exposure-induced increase in S1P was significantly reduced to physiological levels by treatment with a SphK inhibitor. The SphK inhibitor also significantly suppressed light-induced photoreceptor death, both morphologically and functionally (Figure 8 and Figure 9). These results confirmed our hypothesis that S1P synthesis is associated with light reception in the OSs of photoreceptors, and that an increase of S1P above physiological levels causes photoreceptor apoptosis, which results in the pathogenesis of retinal degeneration. Based on these results, the suppressive effect of SphK inhibitors on light-induced retinal degeneration is a potential therapeutic treatment for retinal degeneration.

There were some limitations in our study. First, we used 661W cells in vitro. This immortalized cell line may have different biological signaling and may exhibit different behavior from normal tissue. Second, we examined the role of SphKs in light-induced retinal degeneration only with a SphK inhibitor that was not selective for SphK1 or SphK2. We should have determined which SphK was specifically involved by using knockout mice. Third, we could not fully document the precise relationship between the visual cycle and regulation of the SphK-S1P pathway. More experiments using retinoid cycle inhibitors or knockout mice, such as *ABCA4* knockout mice, are needed to strengthen our hypothesis on the association of SphK1 with atRAL and the visual cycle. Fourth, we used only a LED light as a device to make light-induced retinal degeneration in mice in this present study. Considering retinal degeneration is induced accurately, the LED light we utilized was optimal for our experiments. However, for that reason, we could not determine that the wavelength of light is associated with the enhancement of SphK and S1P, and whether sunlight and the incandescent lamp affect these significant changes. Many previous reports suggested that blue light is involved in retinal degeneration due to high energy and stress, whereas red light is rather involved in retinal protection. A further future study will be needed to investigate which wavelength affects the expression of sphingolipids. Lastly, the concentration of S1P we used to evaluate whether the effect on photoreceptor cell viability is higher than that detected in murine retina or 661W cells. In the present study, we measured the concentration of S1P in whole retinas or whole cells. The expected local enhancement of S1P in OS of the photoreceptor may be higher, so further studies will be required in the future.

In conclusion, we showed, for the first time, the association of S1P and retinal degeneration in photoreceptors during light excitation. The role of SphK1 in cell death was also determined and the inhibition of SphK significantly attenuated retinal damage, both morphologically and functionally. Our findings in photoreceptors, together with the previously mentioned function of the SphK-S1P pathway in photoreceptor cell death and rescue, highlight the potential therapeutic use of a sphingosine 1-phosphate inhibitor in treating retinal degenerative diseases.

## 4. Materials and Methods

### 4.1. Reagents

All-trans-retinal was purchased from Sigma-Aldrich (St. Louis, MO, USA), and the SphK dual inhibitor, SKI-I (ab142209), was purchased from Abcam (Cambridge, UK). S1P was purchased from Enzo Life Sciences (Exeter, UK), and was dissolved in methanol. When using S1P in experiments, methanol was evaporated with nitrogen gas, and S1P was dissolved in bovine serum albumin (lipid free, 40 mg/mL).

### 4.2. Animals

All animal experiments were performed according to the ethical guidelines for animal experimentation of the University of Tokyo (approval number: 医-P18-074, approval date: 23 October 2018) and the ARVO Statement for the Use of Animals in Ophthalmic and Vision Research. BALB/c mice (8-week-old males) were obtained from Japan SLC (Shizuoka, Japan).

### 4.3. Cell Culture

The 661W murine photoreceptor cell line was used in this study. Dulbecco’s modified Eagle’s medium/F12 (Sigma-Aldrich) supplemented with 1% penicillin-streptomycin solution and 10% fetal bovine serum was used as the culture medium. Cultures were incubated at 37 °C in a humidified atmosphere with 5% CO_2_. The medium was replaced every two days. Upon reaching confluency, the cells were serum-starved prior to experiments.

### 4.4. Light Exposure

For the in vitro experiments, the cells were exposed to visible light emitted by a LED (LG-B160C, LEDGO, Derbyshire, UK) after cell starvation. For the in vivo experiments, mice were adapted to dark conditions 24 h prior to light exposure. Mice pupils were treated with 1% cyclopentolate hydrochloride eye drops (Santen Pharmaceuticals, Osaka, Japan) to dilate the pupils before exposure. The mice were then exposed to LED light. To evaluate the effect of the SphK inhibitor on light-induced retinal degeneration, the SphK dual inhibitor SKI-I was used 24 h before light exposure. The mice were anesthetized by intramuscular injection of a mixture of xylazine (10 mg/kg, Bayer, Leverkusen, Germany) and ketamine (50 mg/kg, Daiichi Sankyo Propharma, Tokyo, Japan) in sterile saline, and then SKI-I (500 μM) or vehicle (DMSO) was injected (0.2 μL) intravitreally. After the experiments, the mice were euthanized by an overdose of xylazine and ketamine, and then enucleated.

### 4.5. Measurement of SIP by LC-MS

After the in vitro experiments, the cells were washed twice with phosphate-buffered saline (PBS). Methanol was added to each well, the cells were vortexed for 60 min on ice, and the samples collected. For in vivo experiments, the retinas were isolated from enucleated eyes and immediately stored at −80 °C. The concentration of S1P in each sample was measured using LCMS-8050 (Shimadzu, Kyoto, Japan) and LCMS-8060 (Shimadzu), as previously described [37,38].

### 4.6. qPCR

Total RNA was isolated with TRIzol reagent (Molecular Research Center, Cincinnati, OH, USA), according to the manufacturer’s instructions. cDNA was generated using ReverTra Ace qPCR RT Master Mix with gDNA Remover (Toyobo, Osaka, Japan). The qPCR was performed as previously described [13,14]. Relative mRNA expression values of each sample were normalized to the housekeeping gene of glyceraldehyde-3-phosphate dehydrogenase (GAPDH). The PCR primers are shown in Table 1.

### 4.7. Sphingosine Kinase Assay

After the experiments, the cells were washed twice with PBS. RIPA buffer (Thermo Fisher Scientific, Kanagawa, Japan) containing protease inhibitor cocktail (Roche Diagnostics, Basel, Switzerland) was added to each well. The amount of total protein in each sample was measured using the Pierce BCA Protein Assay (Thermo Fisher Scientific). Sphingosine kinase activity in the same amount of total protein in each sample was measured with the Sphingosine Kinase Activity Assay (Echelon Biosciences, Salt Lake City, UT, USA), according to the manufacturer’s instructions. The luminescence in each sample was calculated using a microplate reader (2030 ARVO X3, Perkin Elmer Japan, Kanagawa, Japan).

### 4.8. Immunohistochemistry

After eyes were enucleated, they were fixed in 4% paraformaldehyde for 24 h, which was followed by immersing in 3% sucrose for two days. Cryosections were made at −20 °C (thickness, 8 μm). Immunohistochemistry was performed as previously described [14]. In brief, the primary antibodies were anti-SphK1 (1:10, Santa Cruz Biotechnology, Dallas, TX, USA), and anti-SphK2 (1:10, Abcam). The secondary antibody was Alexa Fluor 488 anti-rabbit IgG (1:100, Thermo Fisher Scientific). Nuclei were stained with 4′,6-diamidine-2′-phenylindole dihydrochloride (4 μg/mL, PromoCell, Heidelberg, Germany). Images were observed with confocal microscopy (LSM 880 with Airyscan, Zeiss, Tokyo, Japan).

### 4.9. Apoptosis Assay

An Annexin-V-FLUOS Staining Kit (Roche Diagnostics, Mannheim, Germany) was used for evaluating apoptotic cells in vitro, according to the manufacturer’s instructions. In this assay, Hoechst 33342 (Invitrogen, Carlsbad, CA, USA) was also used. To detect apoptotic cells in vivo, fixed cryosections were stained using the Apoptag Fluorescein in Situ Apoptosis Detection Kit (Merck Millipore, Billerica, MA, USA), according to the manufacturer’s instructions. VECTASHIELD^®®^ mounting medium with 4′,6-diamidino-2-phenylindole (Vector Laboratories, Burlingame, CA, USA) was used as the mounting agent. Images were obtained using a BZ-9000 fluorescence microscope (Keyence, Osaka, Japan).

### 4.10. Western Blotting

To investigate the effect of S1P on the protein expression of cleaved caspase-3, Western blotting was performed as previously described [13,14]. In brief, samples containing the same amount of protein were loaded into each well. After electrophoresis with a sodium dodecyl sulfate polyacrylamide gel (Bio-Rad Laboratories, Hercules, CA, USA) and transferring to a polyvinylidene difluoride membrane (Bio-Rad Laboratories), the membrane was incubated with anti-phospho-Akt (Ser473) (1:1000; Cell Signaling Technology, Tokyo, Japan), anti-Akt (1:1000; Cell Signaling Technology), anti-cleaved caspase-3 (1:1000; Cell Signaling Technology), or anti-β-tubulin (1:1000; Wako Pure Chemical Industries, Osaka, Japan) overnight at 4 °C. The blots were captured and analyzed using an ImageQuant LAS 4000 mini-camera system (GE Healthcare, Chicago, IL, USA).

### 4.11. OCT

Five days after light exposure, the anesthetic drugs described in Section 4.4. were injected intramuscularly, and mydriatic eye drops were administrated, followed by OCT (OCT Bi-µ, Kowa, Tokyo, Japan). The thickness of the outer nuclear layer (ONL) was measured using ImageJ software (National Institutes of Health, Bethesda, MD, USA).

### 4.12. ERG

The electroretinogram (ERG) recording used a LS--W (Mayo, Inazawa, Japan) and analyses using LabChart 7 software (ADInstruments, Dunedin, New Zealand). The ERG recordings were performed four days after the light exposure. Mice were adapted to dark overnight. The next day, mice pupils were dilated with the mydriatic eye drops, and the scotopic ERG was performed in the dark under general anesthesia. The flash intensities ranged from 1.5 to 4500 cdms/m^2^. Three trials were averaged for single flash responses.

### 4.13. Statistical Analysis

The statistical significance of differences between two groups was assessed using the unpaired *t*-test, and differences among more than two groups were evaluated by analysis of variance, following multiple comparison tests. The level of significance was set at *p* < 0.05. All calculations were performed using JMP^®®^ Pro software (SAS Institute of Japan, Tokyo, Japan).

## Figures and Tables

**Table 1:** DNA primers used for the quantitative polymerase chain reaction.

Oligos	Forward (5′–3′)	Reverse (3′–5′)
*SphK1*	ACAGTGGGCACCTTCTTTC	CTTCTGCACCAGTGTAGAGGC
*SphK2*	ACCACTTATGAGGAGAATCG	CACCACGTGGTCCATACAGC
*S1P1*	TCATCTGCTGCTTCATCA	CTGCTAATAGGTCCGAGAG
*S1P2*	CTACAATTACACCAAGGAGAC	CAGCACAAGATGATGATGAA
*S1P3*	CACCACCATCCTCTTCTT	ATTGACCTTGTATGCTATGC
*S1P4*	CTCCTGGCTGACATCTTT	TTAATGGCTGAGTTGAACAC
*S1P5*	TCTCTTGCTATTACTGGATGT	TTGGTGAAGGTGTAGATGA
*GAPDH*	AACTTTGGCATTGTGGAAGG	ACACATTGGGGGTAGGAACA
